# Walnut oil increases cholesterol efflux through inhibition of stearoyl CoA desaturase 1 in THP-1 macrophage-derived foam cells

**DOI:** 10.1186/1743-7075-8-61

**Published:** 2011-08-26

**Authors:** Jun Zhang, Jessica A Grieger, Penny M Kris-Etherton, Jerry T Thompson, Peter J Gillies, Jennifer A Fleming, John P Vanden Heuvel

**Affiliations:** 1 Department of Nutritional Sciences, The Pennsylvania State University, University Park, PA, 16802, USA; 2 Center of Excellence in Nutrigenomics, The Pennsylvania State University, University Park, PA, 16802, USA; 3 Department of Veterinary and Biomedical Sciences, The Pennsylvania State University, University Park, PA, 16802, USA; 4 Department of Nutritional Sciences, Institute for Food, Nutrition and Health, Rutgers, The State University of New Jersey, New Brunswick, NJ, 08901, USA

**Keywords:** cholesterol efflux, CRP, FXR, SCD1, walnut oil

## Abstract

**Background:**

Walnuts significantly decrease total and low-density lipoprotein cholesterol in normo- and hypercholesterolemic individuals. No study to date has evaluated the effects of walnuts on cholesterol efflux, the initial step in reverse cholesterol transport, in macrophage-derived foam cells (MDFC). The present study was conducted to investigate the mechanisms by which walnut oil affects cholesterol efflux.

**Methods:**

The extract of English walnuts (walnut oil) was dissolved in DMSO and applied to cultured THP-1 MDFC cells (0.5 mg/mL). THP-1 MDFC also were treated with human sera (10%, v:v) taken from subjects in a walnut feeding study. Cholesterol efflux was examined by liquid scintillation counting. Changes in gene expression were quantified by real time PCR.

**Results:**

Walnut oil treatment significantly increased cholesterol efflux through decreasing the expression of the lipogenic enzyme stearoyl CoA desaturase 1 (SCD1) in MDFC. Alpha-linolenic acid (ALA), the major n-3 polyunsaturated fatty acids found in walnuts, recaptured SCD1 reduction in MDFC, a mechanism mediated through activation of nuclear receptor farnesoid-X-receptor (FXR). Postprandial serum treatment also increased cholesterol efflux in MDFC. When categorized by baseline C-reactive protein (CRP; cut point of 2 mg/L), subjects in the lower CRP sub-group benefited more from dietary intervention, including a more increase in cholesterol efflux, a greater reduction in SCD1, and a blunted postprandial lipemia.

**Conclusion:**

In conclusion, walnut oil contains bioactive molecules that significantly improve cholesterol efflux in MDFC. However, the beneficial effects of walnut intake may be reduced by the presence of a pro-inflammatory state.

**Trial Registration:**

ClinicalTrials.gov: NCT00938340

## Background

Cardiovascular diseases (CVD) are leading causes of morbidity and mortality worldwide. Atherosclerotic thrombus rupture is the major underlying pathologic etiology. To stabilize the arterial plaque and prevent cardiac events, it is critically important to alleviate the peripheral lipid burden. This can be achieved by lowering *de novo *lipogenesis and/or increasing the capacity of reverse cholesterol transport (RCT), a multi-step process transporting extrahepatic lipids to the liver for bile acid secretion.

Numerous studies have shown that nut consumption favorably affects circulating lipids and lipoproteins with LDL-cholesterol (LDL-C) being reduced by 3% to 19% in different populations [1]. While most tree nuts are rich in MUFA, walnuts contain high levels of PUFA, both linoleic acid (LA) and alpha-linolenic acid (ALA). A recent meta-analysis reported that walnut intake consistently reduces total cholesterol and LDL-C in dietary intervention studies [2]. The hypocholesterolemic effects of walnuts are attributed to decreased *de novo *lipogenesis due to their high PUFA content [3]. Thus, walnut PUFA would be predicted to lower the cholesterol burden in atherosclerotic plaques. However, no study to date has evaluated the effects of walnuts on cholesterol efflux.

RCT begins with cholesterol export across the cytoplasm membrane, a process known as cholesterol efflux. Our previous study showed that the omega-3 PUFA ALA significantly decreases cholesterol storage and increases cholesterol efflux in macrophage-derived foam cells by inhibiting the lipogenic enzyme, stearoyl CoA desaturase1 (SCD1) through activation of a nuclear receptor farnesoid-X-receptor (FXR) pathway [4]. SCD1 is an endoplasmic reticulum enzyme that converts saturated fatty acids, palmitic acid and stearic acid, to MUFAs (palmitoleic acid and oleic acid). Relative to their dietary counterparts, endogenously produced MUFAs are preferentially incorporated into triacylglycerols and cholesteryl esters [5]. Due to its critical role in hepatic *de novo *lipogenesis, SCD1 has been proposed as a new drug target for obesity [6] and metabolic syndrome [7]. Manipulation of SCD1 impacts cholesterol efflux as demonstrated in our previous study as well as those of others [8]. However, repressing SCD1 expression by antisense oligonucleotide in atherogenic mouse models showed an inconsistent effect on aorta atherosclerotic plaque formation [9-13]. Thus, it is not clear whether SCD1 could be a drug or dietary target to prevent atherosclerosis progression.

In the present study, we tested the hypothesis that PUFAs, especially n-3 PUFA ALA rich walnut oil would favorably affect cholesterol efflux and SCD1 expression in THP-1 MDFC.

## Methods

### Chemicals

Human LDL, ciprofibrate, rosiglitazone, TO901317, GW4064, β-carotene, γ-tocopherol, β-sitosterol and free fatty acids used in the study were purchased from Sigma-Aldrich; St. Louis, MO. *Z*-Guggulsterone was purchased from EMD Chemicals Inc. (Gibbstown, NJ). GW 501516 and 9-cis retinoic acid (9-cis RA) was purchased from Enzo Life Sciences Inc. (Farmingdale, NY). Purified apoA-I and HDL were purchased from Calbiochem (La Jolla, CA). Rabbit polyclonal anti-SCD1 antibody was a kind gift from Dr. Alan R. Tall (Columbia University). Rabbit polyclonal anti-ACTIN and anti-SHP antibodies were purchased from Santa Cruz Biotechnology Inc. (Santa Cruz, CA). [1α,2α (n)-^3^H] cholesterol was purchased from GE Healthcare Bio-Sciences Corp. (Piscataway, NJ).

### Cell culture

THP-1 (Homo sapiens monocyte) and HEK-293 (Homo sapiens kidney epithelial) cell lines were obtained from the American Type Culture Collection (ATCC; Rockville, MD). Cell culture conditions are as suggested by ATCC.

### Walnut oil preparation in *in vitro *studies

English walnuts were provided by the California Walnut Commission. Shelled whole walnuts were stored at -20 °C before use. Lipid extraction from de-skinned walnut meat was done using a modification of the methods described by Meyer and Terry [14]. The lipid extracts were weighed and dissolved in DMSO to attain a stock concentration of 100 mg/mL. The stock solution was sealed under argon and stored at -20 °C. To minimize the effect of DMSO on gene expression and to maximize oil solubility in the medium, we did a 1:200 dilution of walnut oil stock in the medium to achieve the highest oil treatment concentration as 0.5 mg/mL (DMSO as 0.5%, v:v). This concentration did not cause toxicity as tested by CellTiter Cell Proliferation Assay (Promega; Madison, MI).

### Clinical walnut components acute feeding study

Fifteen subjects with a BMI of 25 to 39 kg/m^2^, LDL ≥ 110 mg/dL and triglycerides (TG) < 350 mg/dL completed a randomized, controlled, four-period, postprandial feeding study. During each of the four visits to the General Clinical Research Center, participants consumed one of the four test diets. The four test diets were randomly provided as 85 g of ground whole walnuts, 34 g of ground de-fatted walnut meat, 51 g of walnut oil or 5.6 g of ground defatted walnut skins. Gram weights of meat, skin and oil fractions were derived from 85 g of whole walnuts. After a baseline blood-draw, participants consumed one of the four walnut components incorporated into diet Jell-OTM over a 10 to 15 min period. At 1, 2, 4, 6 h postprandially, whole blood was drawn. Blood was centrifuged for 15 min to separate sera and stored at -80 °C before use. In between each of the four visits, participants had a 3- to 4-week break, during which they consumed a low antioxidant diet advised by a dietician. The informed consent was written and agreed by all participants. The clinical study conformed to the principles outlined in the Declaration of Helsinki, and was approved and conducted in accordance with the guidelines of the Institutional Review Board of The Pennsylvania State University. The clinical study was registered at clinicaltrials.gov (Identifier: NCT00938340; Study title: Postprandial Effects of Walnut Components Versus Whole Walnuts on Cardiovascular Disease (CVD) Risk Reduction).

### Fatty acid analysis of serum total lipids and HDL phospholipid fraction

All analyses were performed in Lipoprotein Analysis Core Laboratory, Section on Lipid Sciences, Wake Forest University. Samples were saponified in ethanolic KOH and were acidified by the addition of glacial acetic acid. The fatty acids liberated from total lipids were extracted into hexane, which was subsequently evaporated under a stream of nitrogen gas. FAMEs were separated and quantified by gas liquid chromatography (GLC) on a CP Select for FAME capillary column (100 m × 0.25 mm id; Varian, Palo Alto, CA). Each chromatogram was examined to verify the identification of constituent fatty acids. The HDL fraction was collected using an FC 203B Fraction Collector (Gilson Inc.). Lipids were recovered from the HDL using a modified Bligh-Dyer chloroform and methanol extraction procedure [15]. The solvents were evaporated under a stream of nitrogen gas at less than 60°C. The lipid extract was applied as a thin streak to a thin layer chromatography (TLC) plate (Whatman Partisil K6 Silica Gel 60). The silica gel containing the phospholipids (PL) was scraped off the TLC plate and the fatty acid moieties of the PL were converted to more volatile FAMEs *in situ *on the silica gel by the method of Metcalfe *et al *[16]. The FAMEs were extracted into hexane, the solvent evaporated under nitrogen gas at 60°C, dissolved in isooctane, and then transferred to chromatography vials and capped. FAMEs were separated and quantified by GLC as described above.

### Cholesterol efflux

THP-1 human monocytes were differentiated into macrophages by incubating with 100 nM PMA for 48 h in 24-well plates at a density of 3 × 10^5^/well. To induce foam cell formation and equally label intracellular cholesterol pool, cells were loaded with 50 μg/mL oxLDL (prepared as described elsewhere [17]) and ^3^H cholesterol (1 μCi/mL) in normal growth medium. After 24 h, cells were washed twice and treated with walnut oil or 10% (v:v) human sera from each subject of walnut oil group at baseline and 4 h postprandially, respectively, in serum free media. Following a 24-hour treatment, media were collected and centrifuged at 13,200 × *g *for 10 min to remove cell debris. Cells were treated by lysis buffer (5 mM Tris Cl + 0.1% SDS), and media and intracellular tritium (dpm) was measured by liquid scintillation counting.

### RNA extraction, reverse transcription, real time PCR

The detailed procedure was as described elsewhere [18]. Primer sequences were listed in Additional file 1, Table S1 (see supplementary materials online).

### Western blot

THP-1-derived macrophages were seeded in 15 cm^2 ^plates at a density of 5 × 10^6^/plate. Following treatment, cells were treated by lysis buffer as described by Heinemann and Ozols [19]. Lysates were sequentially centrifuged at 800 × *g *and 13,200 × *g*. The protein concentration in the final supernatant was measured by Bio-RAD DC protein assay kit (Bio-RAD Laboratories; Hercules, CA). Total soluble protein was separated on a 12% SDS-PAGE gel and transferred to a PVDF membrane (Immobilon P; Millipore, Bedford MA). All membranes were blocked by 5% non-fat dry milk in TBS + 0.2% Tween 20 (TBS^+^) at 4 °C overnight. The membrane was incubated with primary antibody (anti-SCD1 1:1000; anti-ACTIN 1:500) at room temperature for 2 h. To detect SHP, the membrane was incubated with primary antibody (anti-SHP1 1:200) at 4 °C overnight. After incubation with primary antibody, the blots were washed three times with TBS^+ ^and incubated with horseradish peroxidase-linked secondary anti-rabbit antibodies (1:10,000, 1:5,000 and 1:5,000, respectively) at room temperature for 1 h. Blots were visualized by ECL plus western blot detection kit (GE Healthcare Biosciences; Piscataway, NJ).

### Transfection

Plasmids of human peroxisome proliferator-activated receptor (PPAR)-α/-β/-γ ligand binding domain (LBD), liver-X-receptor (LXR) LBD, and retinoid-X-receptor (RXR-α) LBD were constructed as previously described [20]. Human farnesoid-X-receptor (FXR) and pregnane-X-receptor (PXR) LBDs were constructed using the same methods. Human small heterodimer partner (SHP) expression plasmid pCMX-h*SHP *was a kind gift from Dr. David J. Mangelsdorf (UT Southwestern Medical Center, Dallas). All transfection reactions were cotransfected with *Renilla *luciferase plasmid (pRL-TK) as internal control. HEK293 cells were placed in collagen pre-coated 96-well plate at a density of 2 × 10^4^/well. Each well was transfected with 45 ng cDNA in 30 μL of lipofectamine for 3 h. The well volume was brought up to 100 uL with growth media. Various treatments were then added to the cells and incubated overnight. After 18 to 20 h, the media/treatment was removed and luciferase activities were determined using Promega's Dual Luciferase Assay (Promega; Madison, MI).

### Viral infection

Human SCD1 coding sequence was amplified by PCR using human HepG2 hepatocyte-derived cDNA as a template with primers tailed with *BamHI *and *EcoRI *restriction sites. The PCR product was sub-cloned to lentiviral expression plasmid pCDH-CMV-MCS-EF1-copGFP (System Biosciences; Mountain View, CA). HEK293 cells were transfected with 5 μg pCDH-h*SCD*, 2.4 μg pCMV-VSV-G-RSV-Rev, 2.4 μg pCAG-HIVgp, and 16.5 μl Lipofectamine in 15 cm^2 ^plates. After 5 h, the volume was brought to a total of 10 mL and incubated overnight. The DNA complex was removed the next day and pseudo viruses were expressed by HEK293 cells and secreted in the growth media. After 72 h, media was collected, spun, filtered and applied to infect THP-1 monocyte-derived macrophages with 2 μg/μL of polybrene for 24 h.

### Statistical analyses

General Linear Model (GLM) ANOVA, followed by Tukey post-hoc test, was used to test the difference between treatments (*P *< 0.05). Normality of the data was checked by Anderson-Darling test. The values were expressed as mean ± SEM. All data analyses were performed by Minitab Ver.15 (Minitab Inc., State College, PA) and data plotted by Prism 5.01 (GraphPad Software, Inc., San Diego, CA).

## Results and Discussion

### Walnut oil increases cholesterol efflux and reduces SCD1 expression in THP-1 MDFC

**Figure 1:**
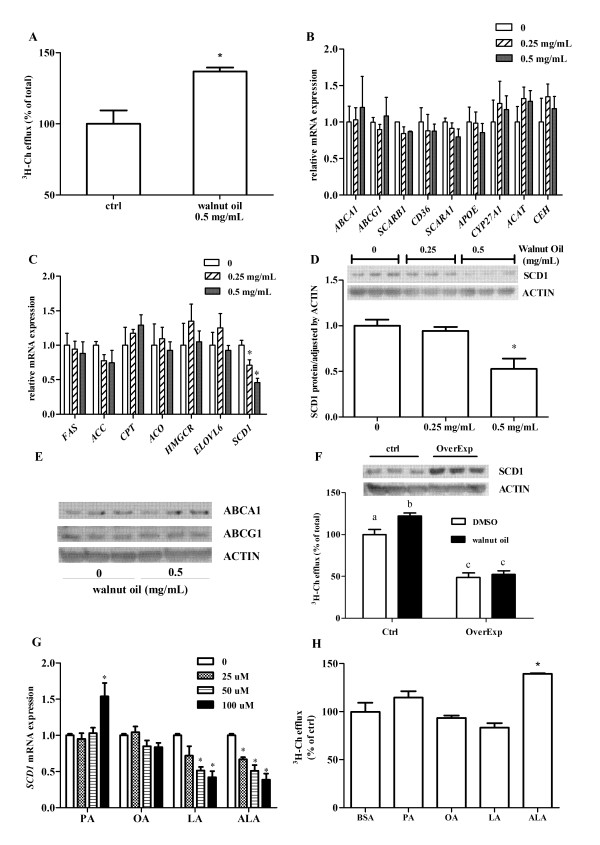
**Walnut oil affects cholesterol transport and storage in THP-1 macrophage-derived foam cells (MDFC)**. (A) Walnut oil increases cholesterol efflux. DMSO treated cells have 2.3% cholesterol efflux (of total ^3^H-Ch). Values of DMSO treated cells are normalized and expressed as 100%. (B) Expression change of genes related with cholesterol transport and storage following walnut oil treatment. Full names of all abbreviation are as described in *Table S1 *footnote. (C) Walnut oil affects gene expression related with lipogenesis and fatty acid oxidation. (D) Walnut oil treatment decreases SCD1 protein. Band intensities were determined by OptiQuant Image analysis software (Packard Instrument Co., Meriden, CT). (E) Effect of walnut oil on ABCA1 and ABCG1 protein levels. (F) Effect of SCD1 on cholesterol efflux. SCD1 overespression is shown by western blot. A total of 60 μg proteins were loaded on each lane of control (empty plasmid) and overexpression groups. Bars not sharing common letters (a, b or c) differ with *P *< 0.05. (G) Fatty acids affect *SCD1 *expression. Fatty acids are conjugated to BSA with a molar ratio of 4:1 modified from method described by Calder et al. [50]. PA: palmitic acid; OA: oleic acid; LA: linoleic acid; ALA: alpha linolenic acid. (H) Fatty acids affect cholesterol efflux. Symbol * in all figure panels indicates a significant difference from respective control with *P *< 0.05. The data presented are means ± SEM of triplicate wells of two independent experiments.

Following walnut oil treatment (0.5 mg/mL), cholesterol efflux was significantly increased by 35%, compared to DMSO control treatment (Figure 1A). Genes related to cholesterol transport and storage were examined following exposure of THP-1 foam cells to walnut oil *in vitro*. Compared to DMSO, walnut oil treatment (0.25 and 0.5 mg/mL) did not significantly affect the expression of genes related to membrane transporter (*ABCA1*, *ABCG1*, SCARB1, *CD36*, *SCARA1*), cholesterol storage (*ACAT*, *CEH*) or secretion (*APOE*, *CYP27A1*) (Figure 1B). Among the key enzymes tested in the lipid synthesis (*FAS*, *ACC*, *HMGCR*, *ELOVL6*) and oxidation (*CPT*, *ACO*) pathways, only *SCD1 *mRNA was significantly reduced in a dose-dependent manner following walnut oil treatment (Figure 1C). Concordant with its effects on transcript levels, SCD1 protein was reduced by approximately 50% following walnut oil (0.5 mg/mL) treatment (Figure 1D) without affecting ABCA1 and ABCG1 proteins, two major membrane transporters mediating cholesterol efflux (Figure 1E). To elucidate the role of SCD1 in cholesterol efflux, this protein was overexpressed in foam cells using lentivirus delivery. Accumulation of SCD1 protein significantly inhibited cholesterol efflux by 52% in DMSO treated cells (Figure 1F). Walnut oil treatment could not restore the cholesterol efflux in cells overexpressing SCD1 (Figure 1F). The regulation of *SCD1 *mRNA by walnut oil was tested further by treatment with the predominant saturated, monounsaturated and polyunsaturated fatty acids found in walnuts. At the highest concentration (100 μM), LA and ALA significantly reduced *SCD1 *expression (- 58% and - 62%, respectively) while the other fatty acids were ineffective (Figure 1G). In contrast, β-carotene (100 nM to 100 μM), γ-tocopherol (100 nM to 100 μM) and β-sitosterol (150 nM to 10 μM) had no effect on *SCD1 *expression in foam cells (data not shown). When THP-1 foam cells were treated with different fatty acids, only ALA elicited an increased level of cholesterol efflux (Figure 1H).

### Walnut oil decreases SCD1 expression by activating FXR in THP-1 MDFC

**Figure 2:**
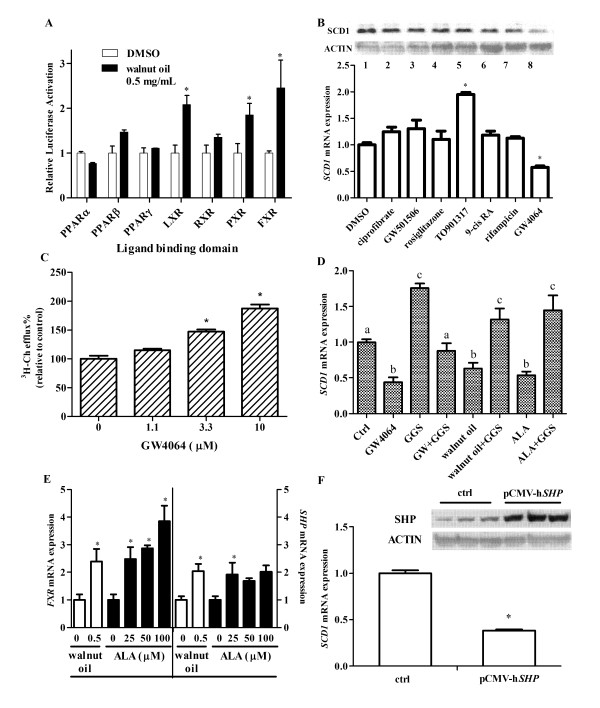
**Walnut oil inhibits SCD1 through a FXR pathway in THP-1 MDFC**. (A) Activation of walnut oil on nuclear receptor ligand binding domain of Dual Luciferase Reporter Assay. PPAR: peroxisome proliferator-activated receptor; LXR: liver-X-receptor; RXR: retinoic-X-receptor; PXR: pregnane-X-receptor; FXR: farnesoid-X-receptor. (B) SCD1 protein (top) and mRNA (bottom) changes following nuclear receptor agonists treatment. SCD1 protein change was detected by Western blot. Treatments are as follows: Lane 1: DMSO control; Lane 2: ciprofibrate 100 μM (PPAR-α agonist); Lane 3: GW501506 500 nM (PPAR-β agonist); Lane 4: rosiglitazone 10 μM (PPAR-γ agonist); Lane 5: TO901317 5 μM (LXR agonist); Lane 6: 9-cis RA 100 nM (RXR agonist); Lane 7: rifamipicin 25 μM (PXR agonist); Lane 8: GW4064 10 μM (FXR agonist). (C) FXR activation increases cholesterol efflux. (D) FXR activation decreases *SCD1 *expression. THP-1 MDFCs were treated with 10 μM GW4064 (FXR agonist), walnut oil (0.5 mg/mL), 100 μM ALA in the presence or absence of 50 μM GGS (FXR antagonist). Bars not sharing common letters are significantly different. (E) Walnut oil and ALA activate *FXR *and increase its target gene SHP expression. (F) Overexpression of SHP inhibits *SCD1 *expression in MDFC. THP-1 derived macrophages were transfected with pCMX-h*SHP *and empty plasmid (control) for 24 h. Symbol * in all figure panels indicates a significant difference from respective control (*P *< 0.05). The data presented are means ± SEM of triplicate wells of two independent experiments.

The regulation of SCD1 expression is due to a complex transcriptional network, including the action of nuclear receptors (NRs), which are targets of fatty acids and various nutrients. Walnut oil treatment significantly increased ligand-dependent activation of human FXR, LXR and PXR, whereas minimal effects were seen for PPARα, β/δ, γ and RXRα (Figure 2A). However, when specific pharmacological agonists of these receptors were examined in THP-1 foam cells, only the FXR agonist GW4064 significantly reduced *SCD1 *mRNA and protein (Figure 2B). In addition, GW4064 increased cholesterol efflux in a dose-dependent manner in these cells (Figure 2C). When the FXR antagonist guggulsterone (GGS) was co-administered with GW4064, walnut oil or ALA, their effect on SCD-1 expression was abolished (Figure 2D). Walnut oil and its predominant n-3 PUFA ALA significantly increased *FXR *mRNA expression (Figure 2E). FXR activation can result in an increased expression of its target gene, small heterodimer partner (*SHP; *Figure 2E), which serves as a repressive transcription factor in the regulation of down-stream gene expression. When SHP was over-expressed in foam cells, *SCD1 *expression was significantly reduced (Figure 2F).

### Following walnut oil intake, human serum rich in ALA significantly decreases SCD1 expression in THP-1 MDFC

**Figure 3:**
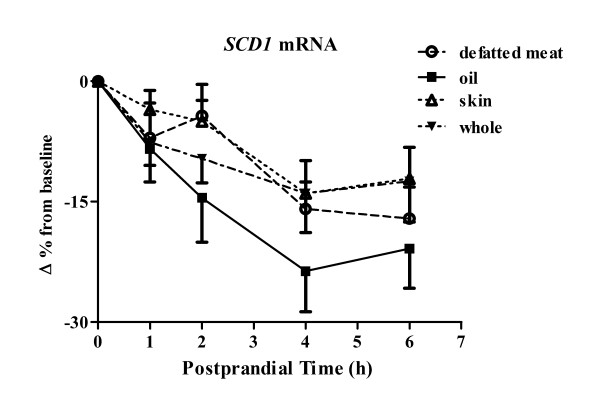
**Human serum affects *SCD1 *expression and cholesterol efflux in THP-1 MDFC**. Human serum affects *SCD1 *expression in THP-1 MDFCs. Samples were taken from 15 subjects consuming different walnut components: whole walnut, walnut oil, defatted walnut meat, and walnut skin. Baseline serum as well as postprandial serum of 1, 2, 4, 6 h were applied as treatment (10%, v:v) for 24 h in serum free media. Real time PCR value of individual serum treated sample is divided by a PCR value of a pooled serum treated sample to minimize the variation between PCR runs and to exclude a non-specific serum effect on gene expression. Value of each postprandial serum treated sample is adjusted by baseline value, which served as a control within the same dietary period. Data were logarithm transformed for analysis. Sera of fifteen participants at baseline and 1, 2, 4, and 6 h postprandially were applied as treatment. N = 75 of each dietary group.

Our *in vitro *data suggests that walnut oil and the n-3 PUFA ALA increases cholesterol efflux in foam cells. The hypothesis tested herein was that bioactive molecules are present in human serum following consumption of walnuts that affect *SCD1 *expression and thus have beneficial effects on cholesterol efflux. *SCD1 *mRNAs were reduced by all postprandial sera taken after consumption of different walnut components with sera of walnut oil group having the greatest *SCD1 *reduction (Figure 3). The *SCD1 *lowering effect between walnut oil and walnut skin groups was significantly different (*P *= 0.001). A significant time effect (*P *< 0.001) on *SCD1 *expression was observed following the intake of all walnut components with the maximal *SCD1 *reduction being achieved 4 to 6 h postprandially (Figure 3). Serum fatty acid analysis was performed to determine the contribution of individual fatty acids to the SCD1 change observed in these cells (Table 1). Following walnut oil intake, saturated fatty acids, myristic acid (14:0) and palmitic acid (16:0), were significantly decreased by 25% and 7%, respectively. Palmitoleic acid (16:1) and oleic acid (18:1) MUFAs both were reduced significantly by 18% and 8%, respectively. Of the major n-6 PUFAs, LA (18:2) was significantly increased (9%) in the postprandial state while gamma-linolenic acid (*γ*18:3) and arachidonic acid (20:4) were decreased (9% and 6%, respectively). Among the n-3 PUFAs evaluated, ALA (α18:3) was significantly increased by 3-fold (197%) after the consumption of walnut oil. Changes in the other n-3 PUFA were not statistically different between sera of baseline and 4 h postprandially. In addition, total n-6 PUFA and n-3 PUFA were increased significantly following walnut oil intake. Specifically, walnut oil intake increased the ratio of ALA/LA by 3-fold, compared with that of baseline.

**Table 1:** Serum fatty acid composition (as percentage of total) of walnut oil group at baseline and 4 h postprandially

Fatty acid	Carbon:	Time		Change
profile	Double-bond	0 h	4 h	4 h vs. 0 h
	14:0	0.93 ± 0.12	0.7 ± 0.08	- 25%^‡^
SFA	16:0	20.5 ± 0.72	19 ± 0.62	- 7.3%^‡^
	18:0	5.83 ± 0.28	5.77 ± 0.23	- 1%

	16:1	2.14 ± 0.23	1.76 ± 0.22	- 17.8%^‡^
MUFA	18:1	21 ± 0.64	19.5 ± 0.64	- 7.8%^‡^

	18:2	34.5 ± 1.37	37.58 ± 1.26	9%^†^
PUFA	*γ*18:3	0.58 ± 0.04	0.53 ± 0.04	- 8.6%^†^
(n-6)	20:4	8.2 ± 0.47	7.67 ± 0.43	- 6.3%^†^

	*α*18:3	0.74 ± 0.06	2.2 ± 0.16	197%^‡^
PUFA	20:5	0.88 ± 0.15	0.83 ± 0.15	- 5.3%
(n-3)	22:5	0.55 ± 0.03	0.52 ± 0.03	- 5.5%
	22:6	1.73 ± 0.16	1.67 ± 0.15	- 3.5%

Total n-6		44.88 ± 1.7	47.3 ± 1.52	5.4%
Total n-3		3.9 ± 0.34	5.1 ± 0.35	30.8%^‡^
n-3/n-6		0.09 ± 0.01	0.11 ± 0.01	22.2%^†^
*α*18:3/18:2		0.02 ± 0.002	0.06 ± 0.004	200%^‡^

Values are expressed as mean (percentage of total fatty acid) ± SEM. MUFA: monounsaturated fatty acid; PUFA: polyunsaturated fatty acid; 14:0 myristic acid; 16:0 palmitic acid; 18:0 stearic acid; 16:1 palmitoleic acid; 18:1 oleic acid; 18:2 linoleic acid; *γ*18:3 gamma-linolenic acid; 20:4 arachidonic acid; *α*18:3 alpha-linolenic acid; 20:5 eicosapentaenoic acid; 20:6 docosapentaenoic acid; 22:6 docosahexaenoic acid. Superscripts ‡: *P *< 0.001; †: 0.001 ≤ *P *< 0.01.

### Categorized by basal CRP level, human sera have different lipidemic responses to dietary interventions

**Figure 4:**
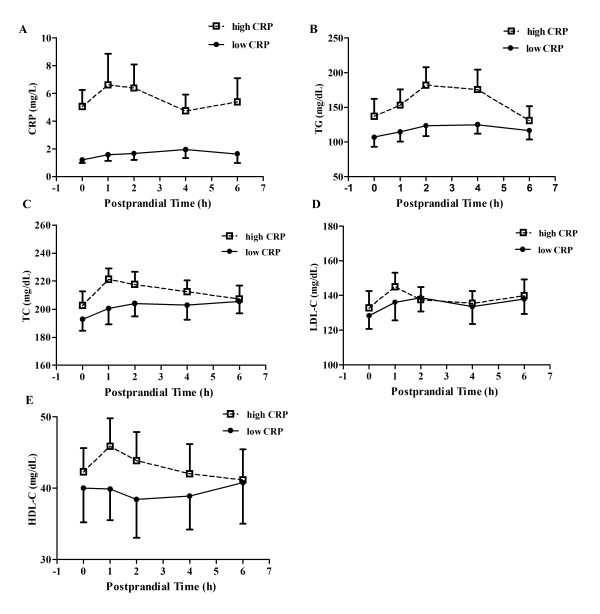
**Categorized by baseline CRP level (2 mg/L as a cutting point), subjects have different serum lipid response following walnut oil intake**. (A) Changes in CRP between low- and high-CRP subgroups. CRP values were expressed as mean (mg/L) ± SEM. Subjects were categorized as low (8 subjects) or high (7 subjects) CRP sub-groups based on the mean of baseline CRP values (*P *< 0.001 between sub-groups). (B) Changes in TG between low- and high-CRP subgroups (*P *< 0.01 between sub-groups). (C) Changes inTC between low- and high-CRP subgroups. (D) Changes in LDL-C between low- and high-CRP subgroups. (E) Changes in HDL-C between low- and high-CRP subgroups. Lipids and lipoproteins were expressed as mean (mg/dL) ± SEM. Open square with dashed line represents the high CRP sub-group. Solid circle with straight line represents the low CRP sub-group.

Study participants were overweight with a mean BMI of 29.7 kg/m^2 ^(BMI = 30 kg/m^2 ^being obese). Five out of fifteen subjects met at least two diagnostic criteria for metabolic syndrome [21]. Multiple metabolic factors of metabolic syndrome, including dyslipidemia, and proinflammatory state, appear to promote the development of atherosclerotic cardiovascular disease [22]. Baseline and postprandial serum lipid parameters (TG, TC, and LDL-C) and inflammatory biomarkers (TNF-α; IL-6, and CRP) are presented in Table 2. Serum CRP from participants who consumed walnut oil ranged through 0.29 mg/L to 17.3 mg/L, with a median of 1.9 mg/L at baseline. Using 2 mg/L CRP as an arbitrary cut point, participants were categorized as low (8 subjects; a mean of 1.6 mg/L) or high (7 subjects; a mean of 5.6 mg/L) CRP sub-groups. Despite a significant difference in CRP levels between the two sub-groups, no time effect on CRP change was observed postprandially (Figure 4A). When data were pooled, serum TG peaked at 4h postprandially with a 26% increase following walnut oil intake, compared to baseline. However, participants in the high CRP sub-group had a significantly higher (36%) postprandial TG response (calculated as area under the curve, AUC), compared with participants in the low CRP sub-group (Figure 4B). TC, LDL-C and HDL-C changes were not significantly different between the two CRP sub-groups (Figure 4C, 4D, 4E).

**Table 2:** Serum lipid and inflammatory biomarker measurement of subjects in walnut oil group

	Lipids				Inflammatory markers		
Time (h)	TG (mg/dL)	TC (mg/dL)	LDL-C (mg/dL)	HDL-C (mg/dL)	TNF-α (pg/mL)	IL-6 (pg/mL)	CRP (mg/L)
0	129.5 (15.1)	197.4 (6.2)	130.5 (5.8)	41.1 (2.9)	1.46 (0.43)	1.28 (0.28)	3 (0.75)
1	136.7 (14.6)	210.2 (7.3)	140.3 (6.6)	42.7 (2.9)	2.05 (0.56)	1.29 (0.32)	3.93 (1.23)
2	158.9 (15.7)*	211 (6.5)	138 (5.2)	41.1 (3.3)	1.5 (0.27)	2.05 (0.54)	4.04 (1.07)
4	163.2 (19.5)*	207.3 (6.6)	134.4 (6.1)	40.3 (3.1)	2.14 (0.7)	1.95 (0.38)	3.25 (0.72)
6	133.3 (14.6)	206.3 (6.1)	138.8 (6.1)	40.9 (3.5)	1.95 (0.66)	2.47 (0.36)	3.38 (0.96)

TG: triacylglycerol; TC: total cholesterol; LDL-C: low-density lipoprotein cholesterol; HDL-C: high-density lipoprotein cholesterol; TNF-α: tumor necrosis factor alpha; IL-6: interleukin 6; CRP: C reactive protein. All values were expressed as mean ± SEM. Inflammatory biomarkers were measured by high sensitive ELISA. The GCRC Core Laboratory at Hershey Medical Center performed the assays for lipids and lipoproteins. LDL-C was calculated using the Friedewald equation [51]. Symbol * indicates a significant difference from baseline.

### The benefits of walnut oil on cholesterol efflux are evident in the low CRP sub-group

**Figure 5:**
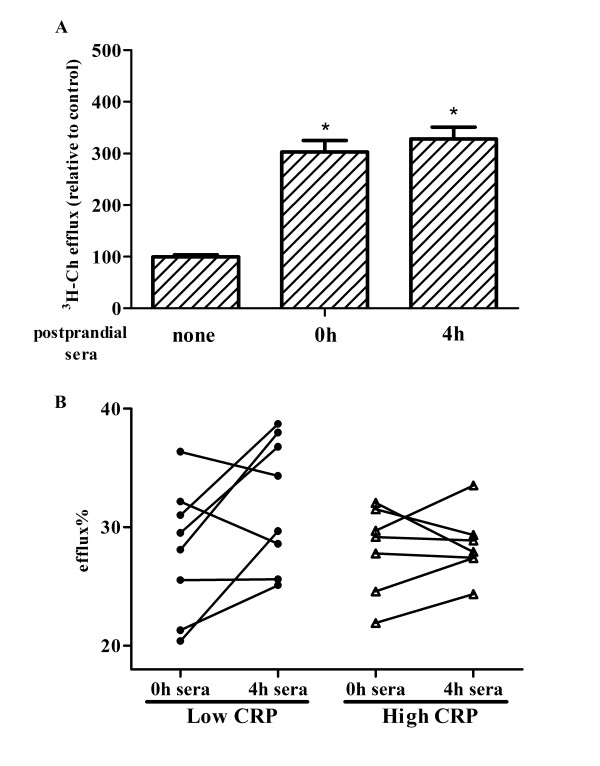
**Human sera of low and high CRP sub-groups differentially affect cholesterol efflux**. (A) Sera of subjects consuming walnut oil affect cholesterol efflux. Following the induction of foam cells, human sera (10%, v:v) of baseline and 4 h postprandially were applied as treatment to induce cholesterol efflux for 24 h. Following efflux period, control cells (incubated in serum-free culture medium only) had a cholesterol efflux of 5.4%. The value is normalized and expressed as 100%. (B) Categorized by baseline CRP (cut point of 2 mg/L), changes of cholesterol efflux following treatment by human sera of walnut oil group. Experiment conditions were as described in Figure 3B. Symbol * indicates a significant difference between control and treatment groups (*P *< 0.05). Results are representative of two independent experiments with a duplicate at each time point (baseline and 4 h postprandially) of each subject.

Serum effects on cholesterol efflux were examined by comparing baseline sera and serum samples collected 4h postprandially in the walnut oil group. The experimental conditions were designed to mimic the *in vivo *response of foam cells exposed to nutrient rich serum during the postprandial state. Compared to non-sera treated cells, both baseline and 4-hour postprandial sera treated cells had significantly increased cholesterol efflux by 3 fold and 3.3 fold, respectively (Figure 5A). However, the difference in efflux between baseline and potprandial sera treated cells was not statistically significant. Interestingly, re-analysis of the efflux data indicated that human sera from the high and low CRP sub-groups had different effects on cholesterol efflux. Sera from the low CRP-subgroup increased cholesterol efflux by 17%, compared to baseline serum treatment, while postprandial sera from the high CRP sub-group did not significantly change cholesterol efflux (Figure 5B). The marked increase of cholesterol efflux following human sera treatment is due to the existence of lipid acceptor HDL in both the baseline and postprandial sera. To rule out the possibility that the increased cholesterol efflux in low CRP sub-group being the functionality change in HDL lipoproteins, i.e. fatty acid enrichment in HDL, HDL lipoprotein in the walnut oil group were isolated from the plasma and a fatty acid analysis of the phospholipid fraction was performed. No significant differences were observed in fatty acids in the HDL phospholipid fraction between baseline and postprandial 4h sera (Table 3).

**Table 3:** Fatty acid composition (as percentage of total) of HDL phospholipid fraction of walnut oil group at baseline and 4 h postprandially

Fatty acid	Carbon:	Time		
profile	Double-bond	0 h	4 h	P value
	16:0	19.6 ± 1.35	20.5 ± 1.4	0.72
SFA	18:0	21.7 ± 0.43	21.8 ± 0.38	0.85

	16:1	0.17 ± 0.05	0.11 ± 0.06	0.48
MUFA	18:1	9.91 ± 0.46	9.8 ± 0.51	0.86

	18:2	23.4 ± 1.13	23.9 ± 1.1	0.74
PUFA	*γ*18:3	0.1 ± 0.02	0.11 ± 0.03	0.94
(n-6)	20:4	21.9 ± 1.18	21.7 ± 1.0	0.89

	*α*18:3	0.21 ± 0.02	0.25 ± 0.02	0.15
PUFA	20:5	1.89 ± 0.43	1.88 ± 0.42	0.98
(n-3)	22:5	2.08 ± 0.17	1.97 ± 0.14	0.63
	22:6	6.47 ± 0.63	6.41 ± 0.6	0.95

Total n-6		51.6 ± 0.9	51.8 ± 0.84	0.85
Total n-3		8.97 ± 0.76	8.85 ± 0.68	0.91
n-3/n-6		0.18 ± 0.02	0.17 ± 0.02	0.88
*α*18:3/18:2		0.009 ± 0.001	0.01 ± 0.001	0.2

Values are expressed as mean (percentage of total fatty acid) ± SEM. Abbreviations are as designated in Table 1 footnote.

### Sera from the low CRP sub-group have more pronounced SCD1 lowering effect without changing ABC transporters in THP-1 foam cells

**Figure 6:**
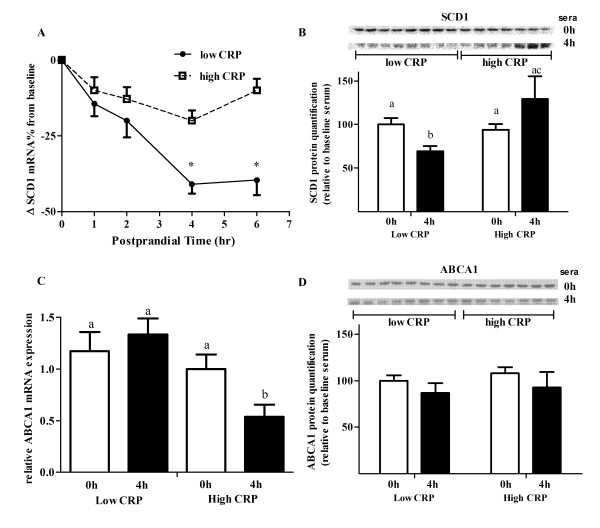
**Human sera of low and high CRP sub-groups differentially affect gene expression**. (A) Changes in *SCD1 *mRNA expression following treatment of human sera from subjects consuming walnut oil. * indicates significant difference from baseline and between CRP sub-groups (*P *< 0.01). (B) Quantification of SCD1 protein. Value of baseline sera treated cells of low CRP sub-group was normalized as 100. Bars not sharing the same letters differ (*P *< 0.05). (C) Changes in membrane transporters *ABCA1 *mRNA following human sera treatment. (D) Changes in ABCA1 proteins categorized by baseline CRP level. In the GLM model, age, gender, BMI, and glucose are served as covariates and ID number as dummy variable.

Our results suggest that the increased level of cholesterol efflux between baseline and postprandial sera from low CRP sub-group was unlikely due to the HDL difference in either concentration (Figure 4E) or composition (Table 3). SCD1, another key regulator in cholesterol efflux as shown in our *in vitro *study, could be significantly affected following sera treatment. Sera from the low CRP sub-group consuming walnut oil did significantly reduce *SCD1 *mRNA expression by 41% and 39%, respectively, at 4 and 6 h postprandially (Figure 6A). There was only a maximum of a 19% reduction in *SCD1 *expression by sera from subjects in the high CRP sub-group, compared to baseline (Figure 6A). Following walnut oil intake, postprandial sera (collected at 4 h) from the low CRP sub-group significantly reduced SCD1 protein level by 30%, compared with that of high CRP sub-group (Figure 6B). In macrophages, the membrane transporters ABCA1 and ABCG1 are the two major ATP binding cassette (ABC) membrane proteins that facilitate cholesterol export [23,24]. Changes in these two transporters could also significantly affect the efficiency of cholesterol efflux. Compared to baseline serum treatment, *ABCA1 *mRNA was reduced following postprandial serum treatment of the high CRP sub-group (Figure 6C). The decreased response of ABCA1 was significantly different from that of the low CRP sub-group. However, ABCA1 protein level was not significantly changed following walnut oil intake (Figure 6D). ABCG1 mRNA and protein were not significantly reduced following postprandial serum treatment and the change was not different between the two CRP sub-groups (data not shown).

In the current study, lipid extracts from walnuts significantly increased cholesterol efflux in foam cells. The findings (Figure 1) suggest that the inhibition of SCD1 by ALA plays a critical role during this process. The results are consistent with several previously published reports, showing that long chain n-3 PUFAs treatment led to an increased level of cholesterol efflux [25-28]. The presence of PUFAs, especially n-3 PUFAs facilitates the free cholesterol movement and incorporation to inner leaflet of plasma membrane [29]. Furthermore, studies have shown that PUFAs can increase membrane fluidity and permeability [30,31], and alter transbilayer sterol localization, resulting in movement of membrane sterols from cytofacial (inner) to exofacial (outer) leaflets [32]. Therefore, ALA-elicited SCD1 inhibition will make free cholesterol more available for the subsequent export across plasma membrane. In addition, over-expression of SCD1 resulted in a decrease in the cholesterol rich region of the membrane [8]. Addition of walnut oil does not restore the cholesterol efflux in the cells over-expressing SCD1 in our study; it is partially due to the non-synergistic activation of the SCD1promoter by various bioactive components in walnut oil. This also may support regulation of SCD1 expression being important to walnut oil's ability to affect cholesterol efflux. Several studies [33-36] indicated that PUFA could decrease cholesterol efflux, possibly through degradation of ABCA1 via protein kinase C delta pathway [37]. In the present study, walnut oil treatment did not result in significant changes in ABC transporter proteins. Our previous study also showed that ALA increased cholesterol efflux without affecting membrane ABC proteins in macrophage-derived foam cells [4]. Knocking down SCD1 through siRNA reproduced the increased cholesterol efflux without changing ABC transporters in foam cells [4]. Thus, the increased cholesterol efflux induced by walnut oil is unlikely due to the regulation of membrane ABC transporters.

Lipid extracts from walnut components significantly activated FXR to a greater extent than other tested nuclear receptors. FXR plays a critically important role in bile acid synthesis, secretion and re-absorption, and is expressed at a high level in liver and intestine. Gene knockout studies demonstrated an unexpected role for FXR in lipid metabolism, where activation results in hypolipidemia [38]. However, a direct effect of FXR on atherosclerosis is still controversial [39-41]. Our *in vitro *studies presented herein demonstrate that walnut oil, especially its PUFA fraction increases cholesterol efflux. This effect is mediated by a decrease of SCD1 through activation of FXR pathway. This suggests that the action of FXR in our model is atheroprotective.

To extrapolate the *in vitro *findings to humans, serum samples taken from subjects consuming different walnut components were applied as a treatment to cultured THP-1 foam cells. Sera from individuals consuming walnut components (whole walnut, oil, walnut skin and defatted walnut meat) all decreased *SCD1 *expression in THP-1 foam cells. There was a greater reduction in SCD1 expression in serum samples from the walnut oil group versus the whole walnut group. This could, in part, be due to a different absorption rate that relates to the physical form of the food [42]. Compared to whole walnuts, walnut oil would have a faster absorption rate that would result in a greater effect on gene regulation. Walnut skins are higher in antioxidants (23 mmol/100 g), compared to most other commonly consumed tree nuts [43]. Chen *et al *found that flavonoids and polyphenolics from almond skin extracts enhanced the resistance of LDL to copper-induced oxidation both *in vitro *and *in vivo *[44]. This suggests that walnut skins decreased *SCD1 *expression by regulating oxidative products generation and release into the circulation. However, it still is not clear what the exact mechanisms are for *SCD1 *lowering following defatted walnut meat intake.

The postprandial sera-induced cholesterol efflux is unlikely due to a functionality change in HDL since there was no change in HDL concentration or relative fatty acid composition change in this fraction between baseline and the postprandial time points or between the low or high CRP sub-groups. In contrast, a three-fold increase of ALA in the total lipid extract of sera was observed following walnut oil intake. In the postprandial state, the increased level of ALA is presumably in triglyceride rich lipoproteins, such as VLDL or chylomicrons rather than HDL particles. The influx of nutrients from the GI tract will markedly increase lipoprotein lipase activity, which will release free fatty acid into the blood stream. These newly released fatty acids, including ALA, could affect cholesterol efflux in macrophages. Fatty acid enrichment and lipid exchange between HDL and other lipoproteins typically are regulated by lecithin-cholesterol acyltransferase (LCAT) and cholesteryl ester transfer protein (CETP). A 6-hour observation period may not have been sufficient to achieve the maximal LCAT and CETP activities or obtain the maximal enrichment of ALA in HDL to induce cholesterol efflux. Thus, long-term feeding studies are needed to evaluate the effects of walnuts on HDL functionality changes.

In our study, sera from subjects consuming walnuts decreased *SCD1 *expression and increased cholesterol efflux. Dyslipidemia and systemic inflammation are major risk factors leading to cardiac events and exacerbating atherosclerosis development and progression. A higher CRP level is a marker of a pro-inflammatory state and is highly correlated with increased risks of metabolic syndrome and cardiovascular diseases [22]. Results from several studies demonstrated that an elevated inflammatory state, such as increased levels of CRP [45], TNF-α and IL-1β [46,47] impairs cholesterol efflux. Furthermore, inflammatory mediators increase SCD1 activity in mouse macrophages [48] and in a longitudinal study in humans [49]. In the present study, although *SCD1 *mRNA was slightly decreased by serum from the high CRP sub-group, this did not translate into any significant improvement in efflux presumably due to the higher levels of circulating inflammatory mediators.

## Conclusion

The studies presented herein demonstrated that lipid-rich walnut oil significantly reduced SCD1 expression as well as increased cholesterol efflux in macrophage-derived foam cells, which will be of benefit for atherosclerosis regression.

## List of Abbreviations

SCD1: stearoyl CoA desaturase 1; FXR: farnesoid X receptor; CRP: C reactive protein;

## Competing interests

All authors declare no competing interests.

## Authors' contributions

JZ conducted most of the cell culture experiments, collected and interpreted the data and wrote the manuscript. JAG led the clinical dietary feeding study. PMKE designed and coordinated the clinical study. JTT conducted the transfection and viral infection experiments. PJG interpreted clinical data and gave critical revision. JAF helped design the clinical study and screened participants at baseline. JPVH designed all the cell culture experiments, interpreted the data and gave critical review. All authors have read and approved the final manuscript.
